# Longitudinal association between adiposity changes and lung function deterioration

**DOI:** 10.1186/s12931-023-02322-8

**Published:** 2023-02-07

**Authors:** Youngmok Park, Jiyoung Kim, Young Sam Kim, Ah Young Leem, Jinyeon Jo, Kyungsoo Chung, Moo Suk Park, Sungho Won, Ji Ye Jung

**Affiliations:** 1 Division of Pulmonary and Critical Care Medicine, Department of Internal Medicine, Severance Hospital, Yonsei University College of Medicine, 50-1 Yonsei-ro, Seodaemun-gu, Seoul, 03722 South Korea; 2 Yonsei University College of Medicine, Seoul, South Korea; 3 Department of Public Health Sciences, School of Public Health, Seoul National University, Seoul, South Korea; 4 Institute of Health and Environment, Seoul National University, Seoul, South Korea; 5 RexSoft Corps, Seoul, South Korea; 6 Suwa Central Hospital, Chino-shi, Nagano, Japan

**Keywords:** Abdominal obesity, Adiposity, Body composition, Spirometry, Waist–hip ratio

## Abstract

**Background:**

The longitudinal relationship between adiposity and lung function is controversial. We aimed to investigate the long-term association between adiposity changes and lung function in a middle-aged general Asian population.

**Methods:**

In total, 5011 participants (average age, 54 years; 45% men) were enrolled from a community-based prospective cohort. During the follow-up period (median 8 years), both spirometry and bio-electrical impedance analysis were performed biannually. Individual slopes of the fat mass index (FMI; fat mass divided by the square of height in meters) and waist-to-hip ratio (WHR) were calculated using linear regression analysis. Multivariate linear mixed regression analysis was used to determine the long-term association between adiposity changes and lung function.

**Results:**

The FMI was inversely associated with forced vital capacity (FVC) (estimated: − 31.8 mL in men, − 27.8 mL in women) and forced expiratory volume in 1 s (FEV_1_) (estimated: − 38.2 mL in men, − 17.8 mL in women) after adjusting for baseline age, height, residential area, smoking exposure (pack-years, men only), initial adiposity indices, and baseline lung function. The WHR was also inversely associated with FVC (estimated = − 1242.2 mL) and FEV_1_ (estimated = − 849.8 mL) in men. The WHR-increased group showed a more rapid decline in lung function than the WHR-decreased group in both the fat-gain and fat-loss groups.

**Conclusion:**

Adiposity was associated with the long-term impairment of lung function. Central obesity was the main driver of lung function impairment in the middle-aged general Asian population, regardless of fat mass changes.

**Supplementary Information:**

The online version contains supplementary material available at 10.1186/s12931-023-02322-8.

## Background

The global prevalence of obesity has doubled during the past three decades [1]. In 2015, approximately 39% of the world’s population was estimated to be overweight or have obesity [2]. The condition has been shown to be a risk factor for various lung diseases, especially those associated with the deterioration of pulmonary function [3]. Consequently, obesity poses a substantial health burden.

Previous studies have explored the longitudinal association between adiposity and lung function using assessment of bodyweight or body mass index (BMI) [4–7]. Nevertheless, the single use of bodyweight or BMI as a measure of obesity can be misleading as weight or BMI are poor measures of adiposity, resulting in inconsistent conclusions [8]. A few cross-sectional studies have investigated the relationship between lung function and body composition parameters, including body fat mass and its distribution, by employing bio-electrical impedance analysis (BIA) or dual-energy X-ray absorptiometry. Body fat distribution has a stronger association with lung function than bodyweight or BMI [9, 10]. Further, the effects of body fat on different sites have shown comparable effects on respiratory function [10, 11].

However, it remains unclear how long-term body composition changes are related to lung function impairment. Few studies have tried to elucidate this long-term association; however, several limitations were observed due to specific age ranges (young/old), small sample size, and measurements at only two timepoints (the beginning and end) [12–14].

In this study, we examined the long-term association between adiposity and lung function changes using BIA in a large community-based cohort. Anthropometric and spirometry data were collected repeatedly during the follow-up period, and we categorized the study population according to changes in the fat mass index (FMI) and waist-to-hip ratio (WHR). To the best of our knowledge, this comprehensive study is the first to use the individual slope of adiposity changes to elucidate the association between adiposity and respiratory function in a middle-aged Asian population.

## Methods

### Study population

**Fig. 1:**
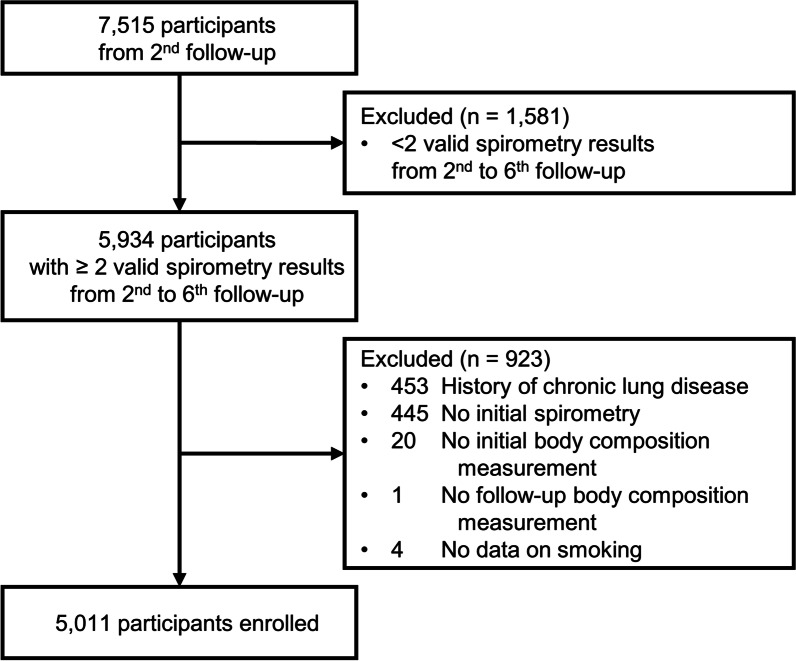
Flowchart of the participant selection process

The participants in this study were recruited from the Ansan-Ansung cohort, an ongoing population-based epidemiologic survey. The cohort is a part of the National Genome Research Institute-supported Korean Genome and Epidemiology Study, a large community-based epidemiologic survey to investigate chronic diseases among South Koreans [15, 16]. The cohort comprises a population-based sample of male and female South Koreans, aged 40–69 years, from two different sites: Ansan, an urban community with a population of 555,000 residents, and Ansung, a rural community with 133,000 residents [16]. The participants from the baseline (2001–2002) were followed up biannually to the 6th follow-up (2013–2014). Detailed numbers of participants in each follow-up cohort are provided in Additional file 1: Table S1. Quality-controlled spirometry results were available from the 2nd follow-up; therefore, 7515 participants in the 2nd follow-up were initially screened, and 5934 individuals with ≥ 2 valid spirometry results were identified between the 2nd and 6th follow-ups (Fig. 1). The following participants were excluded: (1) those with chronic lung diseases, such as chronic obstructive pulmonary disease, asthma, and bronchiectasis; (2) those without spirometry data in the 2nd follow-up; (3) those who did not undergo BIA in the 2nd follow-up; (4) those who did not undergo follow-up BIA, and (5) those with an invalid smoking history. Finally, 5011 participants were enrolled in this study.

### Spirometry

Lung function was evaluated using spirometry (Vmax-2130, Sensor-Medics, Yorba Linda, CA) at all baseline and follow-up visits. Each test was performed according to standardized protocols of the American Thoracic Society [17]. We used Morris and Polgar’s equation as a reference for normal lung function [18].

### Body composition measurement and adiposity index

Anthropometric data were collected using multi-frequency BIA (InBody 3.0, Biospace, Seoul, South Korea) [19]. The multi-frequency BIA device measures the impedance of body tissues by subjecting the body to imperceptible electrical signals using an eight-point tactile electrode system. Previous studies have demonstrated that multi-frequency BIA can produce reliable body composition estimates, which are compatible with those measured by dual-energy X-ray absorptiometry [20, 21].

We used the FMI and WHR as adiposity indices. The fat mass measured using multi-frequency BIA was divided by the square of height in meters and designated as the FMI (kg/m^2^) [13]. We calculated individual slopes of FMI changes during the follow-up using linear regression, and participants were categorized into two groups: “fat-gain,” with increased FMI, and “fat-loss,” with decreased FMI. Waist and hip circumferences were measured three times at every visit. The average waist circumference divided by the average hip circumference was defined as the WHR. Abdominal obesity was defined as WHR ≥ 0.90 for men and ≥ 0.80 for women [22]. Individual slopes of WHR changes during the follow-up were calculated using linear regression, and we divided the participants into three groups: WHR-increased (slope of WHR change is upper 30% of participants), WHR-decreased (slope of WHR change is lower 30% of participants), and WHR-stable groups (slope of WHR change is median 40% of participants, which includes zero-degree slope). We further categorized the fat-loss and fat-gain groups in the study population into the WHR-decreased, -stable, and -increased groups. None of the participants FMI or WHR remained constant.

### Statistical analyses

Continuous variables were compared using Student’s *t*-test or the Mann–Whitney U test; categorical variables were analyzed using the Pearson χ^2^ test or Fisher’s exact test. In three group comparison, one-way analysis of variance was used. Pearson correlation analyses were used to test associations between these variables. Linear regression analyses for each participant were performed to calculate the individual slopes of FMI and WHR during the follow-up. The signs of slopes were utilized to identify fat-gain (slope of FMI change > 0) and WHR-increased group (slope of WHR change is upper 30% of participants). The longitudinal associations between lung function and adiposity changes were evaluated using multiple linear mixed regression analysis after adjusting for age, height, residential area, follow-up duration, initial adiposity indices, interaction between age and adiposity indices (if needed), and initial lung function. Baseline lung function was adjusted in the models because it might affect the degree of lung function decline, especially in long-term follow-up of 8 years, and because we tried to absorb effects from adiposity indices up to baseline [23]. Most men were smokers, and we additionally adjusted for smoking status amount in the regression model of men. However, as there were few female smokers, we excluded ever-smoker women from the regression analyses. The participant identification number was utilized as a random effect to adjust the similarity between the multiple observations from the same participant. We calculated beta parameters using restricted maximum likelihood methods, and *P*-value with the Wald test. All statistical analyses were performed with R software v4.0.2 (The R Foundation for Statistical Computing, Vienna, Austria), and multiple linear mixed regression analysis was conducted with the lme4 package. A two-tailed *P-*value of < 0.05 was considered statistically significant for all analyses.

## Results

### Baseline characteristics

Table 1 shows the baseline characteristics and follow-up information of the study population stratified by sex. The mean age was 54.0 ± 7.9 years, and 45.0% of the participants were men. The baseline characteristics varied based on their sex, including adiposity indices. Men had a lower BMI (24.5 kg/m^2^ vs. 24.8 kg/m^2^, *P* = 0.005) and a higher proportion of ever-smokers (73.6% vs. 2.5%, *P* < 0.001) than women. The baseline FMI was lower in men than in women (5.3 kg/m^2^ vs. 7.8 kg/m^2^, *P* < 0.001), although the proportions of participants with fat gain were similar (66.1% vs. 64.8%, *P* = 0.284). Initial abdominal obesity was less frequent in men than in women (62.2% vs. 88.5%, *P* < 0.001).

**Table 1:** Baseline characteristics and follow-up data of study participants

	Total (N = 5011)	Men (n = 2255)	Women (n = 2756)	*P-*value
Age, years	54.0 ± 7.9	53.1 ± 7.5	54.8 ± 8.3	< 0.001
Height, cm	160.2 ± 8.6	167.4 ± 5.7	154.3 ± 5.5	< 0.001
BMI, kg/m^2^	24.7 ± 2.9	24.5 ± 2.7	24.8 ± 3.1	0.005
Ever-smoker	1727 (34.5)	1659 (73.6)	68 (2.5)	< 0.001
Smoking exposure, pack-year	8.6 ± 15.6	18.7 ± 18.6	0.3 ± 2.7	< 0.001
Residential area—rural	2136 (42.6)	838 (37.2)	1298 (47.1)	< 0.001
Residential area—urban	2875 (57.4)	1417 (62.8)	1458 (52.9)	
Adiposity index				
FMI, kg/m^2^	6.7 ± 2.3	5.3 ± 1.7	7.8 ± 2.2	< 0.001
Fat gain^a^	3277 (65.4)	1490 (66.1)	1787 (64.8)	0.284
WHR	0.91 ± 0.07	0.92 ± 0.06	0.90 ± 0.08	< 0.001
Abdominal obesity^b^	3303 (66.0)	1399 (62.2)	2434 (88.5)	< 0.001
WHR-increased participants^a^	1503 (30.0)	676 (30.0)	827 (30.0)	> 0.999
Lung function				
FVC, L	3.57 ± 0.84	4.26 ± 0.63	3.00 ± 0.50	< 0.001
FVC, % predicted	104.4 ± 12.5	101.8 ± 11.8	106.5 ± 12.7	< 0.001
FEV_1_, L	2.86 ± 0.65	3.36 ± 0.52	2.45 ± 0.42	< 0.001
FEV_1_, % predicted	112.9 ± 14.8	108.5 ± 13.2	116.6 ± 15.1	< 0.001
FEV_1_/FVC, %	80.3 ± 4.8	78.9 ± 4.8	81.5 ± 4.5	< 0.001
Measurement				
Follow-up duration, year	8 (6–8)	8 (6–8)	8 (8–8)	0.009
Spirometry, times	4 (3–5)	4 (3–5)	4 (3–5)	0.166
BIA, times	5 (4–5)	5 (4–5)	5 (4–5)	0.017

Data are presented as numbers (%), means ± standard deviations, or medians (interquartile ranges), unless otherwise indicated

*BIA* bio-electrical impedance analysis, *BMI* body mass index, *FEV*_*1*_ forced expiratory volume in 1 s, *FMI* fat mass index, *FVC* forced vital capacity, *WHR* waist-to-hip ratio

^a^Individual changes in the FMI and WHR during follow-up were calculated with linear regression analysis. Participants with a slope of FMI change > 0 were classified under the fat-gain group, and those with a slope of WHR change is upper 30% of participants were classified into the WHR-increased group

^b^Abdominal obesity was defined by WHR values of ≥ 0.90 and ≥ 0.80 for men and women, respectively

The study participants were followed up for a median of 8 (interquartile range, 6–8) years, with spirometry and BIA being continuously performed for a median of 4 (interquartile range, 3–5) and 5 (interquartile range, 4–5) times, respectively.

### Cross-sectional association between adiposity and lung function

**Fig. 2:**
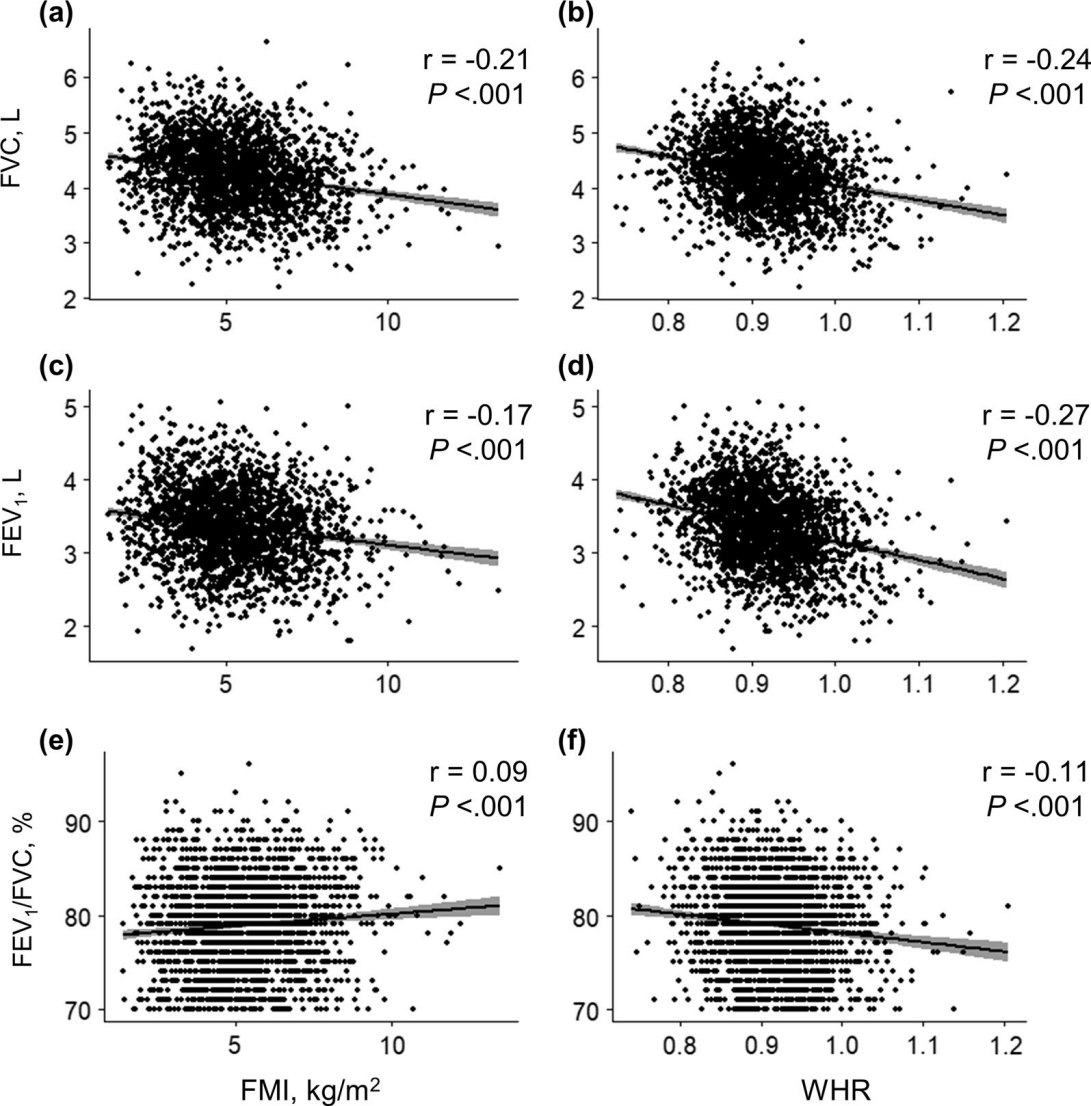
Association between adiposity indices and baseline lung function in men. Pearson correlations between **a** FVC and FMI (r = − 0.21, *P* < 0.001), **b** FVC and WHR (r = − 0.24, *P* < 0.001), **c** FEV_1_ and FMI (r = − 0.17, *P* < 0.001), **d** FEV_1_ and WHR (r = − 0.27, *P* < 0.001), **e** FEV_1_/FVC and FMI (r = 0.09, *P* < 0.001), and **f** FEV_1_/FVC and WHR (r = − 0.11, *P* < 0.001). *FEV*_*1*_ forced expiratory volume in 1 s, *FMI* fat mass index, *FVC* forced vital capacity, *WHR* waist-to-hip ratio

**Fig. 3:**
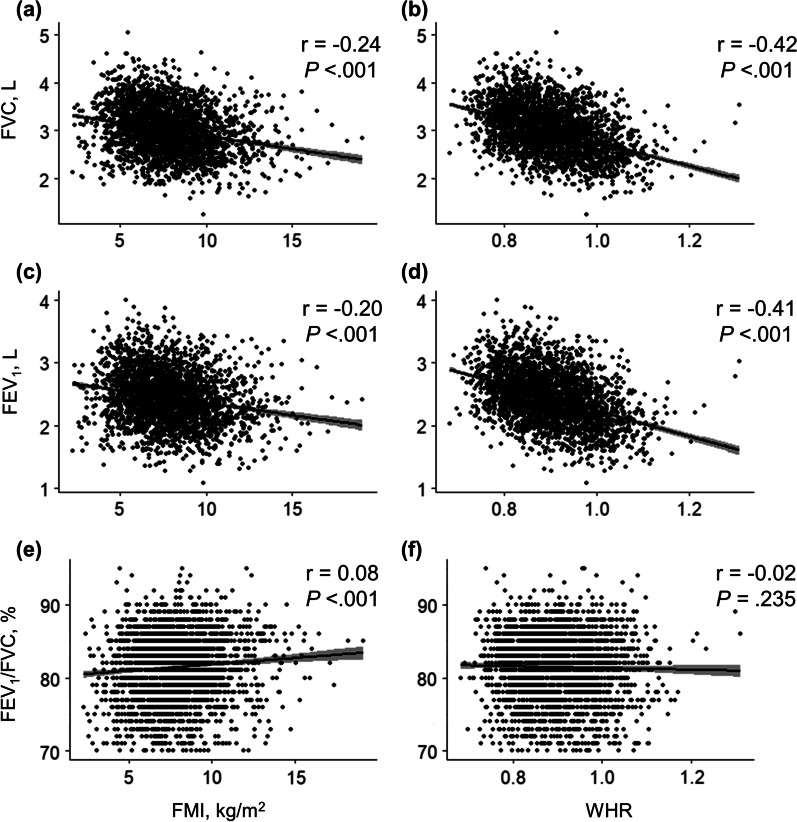
Association between adiposity indices and baseline lung function in women. Pearson correlations between **a** FVC and FMI (r = − 0.24, *P* < 0.001), **b** FVC and WHR (r = − 0.42, *P* < 0.001), **c** FEV_1_ and FMI (r = − 0.20, *P* < 0.001), **d** FEV_1_ and WHR (r = − 0.41, *P* < 0.001), **e** FEV_1_/FVC and FMI (r = 0.08, *P* < 0.001), and **f** FEV_1_/FVC and WHR (r = − 0.02, *P* = 0.235). *FEV*_*1*_ forced expiratory volume in 1 s, *FMI* fat mass index, *FVC* forced vital capacity, *WHR* waist-to-hip ratio

Figures 2 and 3 show the cross-sectional association between lung function and adiposity indices at baseline in men and women, respectively. FMI and WHR were inversely associated with forced vital capacity (FVC) (men: r = − 0.21 with FMI, r = − 0.24 with WHR; women: r = − 0.24 with FMI, r = − 0.42 with WHR; all *P* < 0.001) and forced expiratory volume in 1 s (FEV_1_) (men: r = − 0.17 with FMI, r = − 0.27 with WHR; women: r = − 0.20 with FMI, r = − 0.41 with WHR; all *P* < 0.001). In men, WHR was also negatively associated with FEV_1_/FVC ratio (r = − 0.11, *P* < 0.001).

### Longitudinal association between adiposity and lung function changes

We used multiple linear mixed models to determine possible long-term associations between adiposity indices and lung function. Table 2 demonstrates the association of FMI and WHR with lung function parameters. The FMI was associated with a decrease in FVC (estimated = − 31.8 mL in men; − 27.8 mL in women) and FEV_1_ (estimated = − 38.2 mL in men; − 17.8 mL in women) (all *P* < 0.01). In men, WHR showed an inverse association with FVC (estimated = − 1242.2 mL) and FEV_1_ (estimated = − 849.8 mL) (all *P* < 0.001). Full models of men are presented in Additional file 2: Table S2; smoking enhanced the deterioration of FEV_1_ and FEV_1_/FVC in men. Age and residential area also affected the lung function decline.

**Table 2:** Multiple linear mixed regression analysis for long-term associations between adiposity indices and lung function

	FVC, mL			FEV_1,_ mL			FEV_1_/FVC, %		
	Estimate (95% CI)	SE	*P*-value	Estimate (95% CI)	SE	*P*-value	Estimate (95% CI)	SE	*P*-value
Men^a^									
FMI, kg/m^2^	− 31.8 (− 52.3, − 11.3)	10.5	0.002	− 38.2 (− 55.4, − 21.0)	8.8	< 0.001	− 0.04 (− 0.35, 0.26)	0.15	0.775
WHR	− 1242.2 (− 1811.3, − 672.4)	290.7	< 0.001	− 849.8 (− 1323.7, − 373.5)	241.8	< 0.001	8.30 (− 0.14, 16.77)	4.31	0.054
Women^b^									
FMI, kg/m^2^	− 27.8 (− 38.6, − 17.0)	5.5	< 0.001	− 17.8 (− 26.9, − 8.8)	4.6	< 0.001	0.27 (0.09, 0.45)	0.09	0.004
WHR	− 159.3 (− 452.1, 133.4)	2.7	0.902	− 69.4 (− 313.2, 174.7)	124.5	0.577	4.96 (− 0.02, 9.95)	2.54	0.051

*FEV*_*1*_ forced expiratory volume in 1 s, *FMI* fat mass index, *FVC* forced vital capacity, *SE* standard error, *WHR* waist-to-hip ratio

^a^Men: adjusted for age, height, residential area, follow-up duration, smoking exposure in pack-years, initial adiposity indices, interaction between age and adiposity indices, and initial lung function

^b^Non-smoking women: adjusted for age, height, residential area, follow-up duration, initial adiposity indices, interaction between age and adiposity indices, and initial lung function

### Longitudinal association between WHR and lung function changes in the fat-loss and fat-gain groups

During the follow-up period, we further categorized the fat-loss and fat-gain groups in the study population into the WHR-decreased, -stable, and -increased groups (Additional file 3: Table S3, Additional file 4: Table S4). The proportions of abdominal obesity at baseline were different among changes of WHR in men (fat-loss: 70.8% vs. 60.4% vs. 50.8%, *P* < 0.001; fat-gain: 76.8% vs. 66.8% vs. 46.4%, *P* < 0.001) and women (fat-loss: 97.2% vs. 88.3% vs. 83.2%, *P* < 0.001; fat-gain: 97.7% vs. 89.8% vs. 77.0%, *P* < 0.001).

**Fig. 4:**
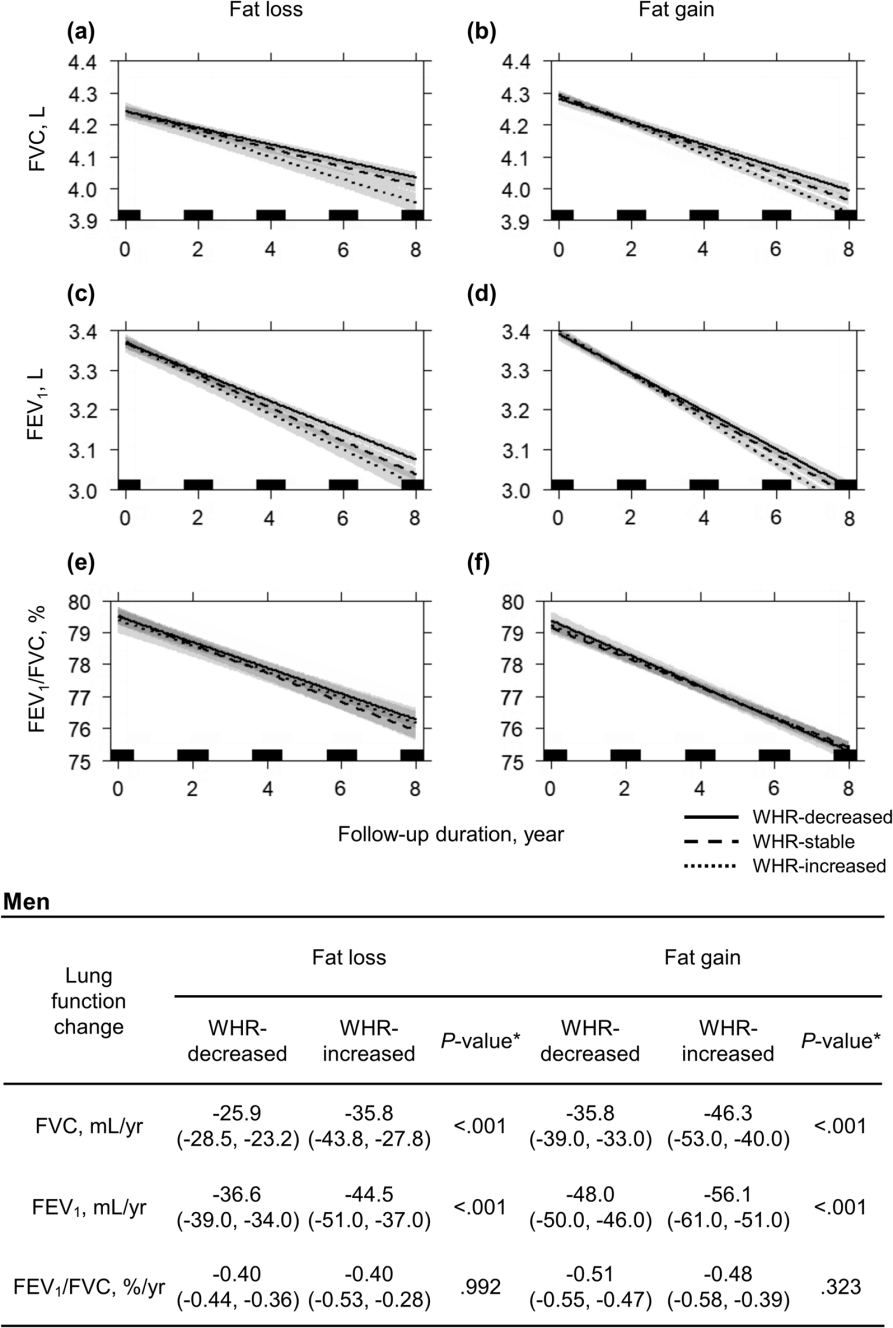
Multiple linear mixed regression analysis for lung function decline according to adiposity changes in men. Lung function declines were compared between the WHR-decreased and -increased groups in fat- loss and fat-gain group, respectively. Decline of **a** FVC in the fat-loss group, **b** FVC in the fat-gain group, **c** FEV_1_ in the fat-loss group, **d** FEV_1_ in the fat-gain group, **e** FEV_1_/FVC in the fat-loss group, and **f** FEV_1_/FVC in the fat-gain group. Individual changes in FMI during follow-up were calculated with linear regression analysis. Participants with a slope of FMI change < 0 were classified under the fat-loss group, and those with a slope > 0 were classified under the fat-gain group. No participant had a zero-degree slope of FMI change throughout the study period. Individual changes in WHR during follow-up were calculated with linear regression analysis. Participants with a lower 30% of WHR change were designated to WHR-decreased group, and those with an upper 30% of WHR change were to WHR-increased group. WHR-stable group comprised the median 40% of participants, which included a zero-degree slope. Results were adjusted for age, height, residential area, follow-up duration, smoking exposure (pack-years), initial lung function, initial FMI, and initial WHR. The gray shadow and numbers in parentheses represent 95% confidence intervals. *P-value between WHR-decreased and -increased group. *FEV*_*1*_ forced expiratory volume in 1 s, *FMI* fat mass index, *FVC* forced vital capacity, *WHR* waist-to-hip ratio

**Fig. 5:**
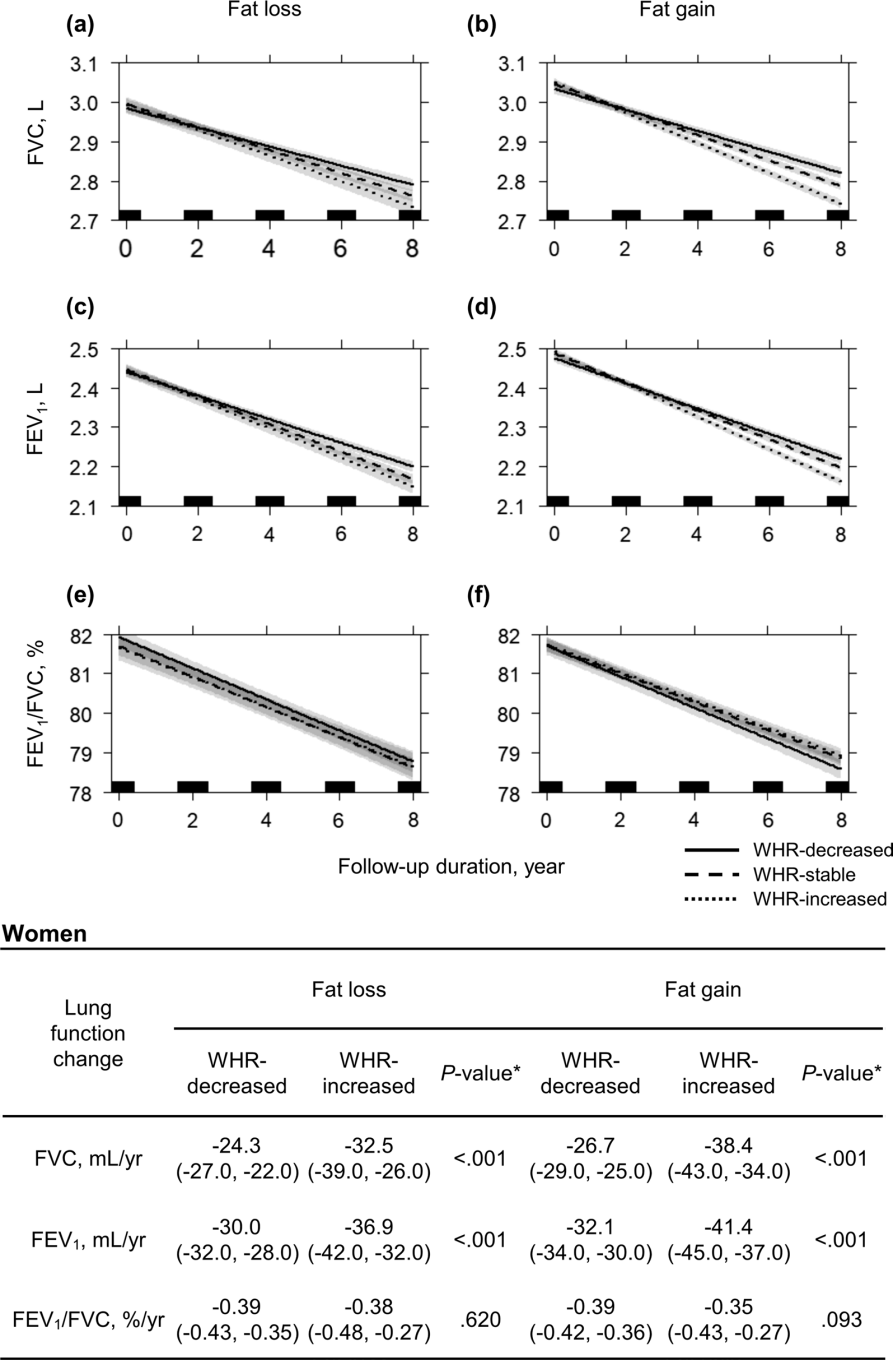
Multiple linear mixed regression analysis for lung function decline according to adiposity changes in women. Lung function declines were compared between the WHR-decreased and -increased groups in fat-loss and fat-gain group, respectively. Decline of **a** FVC in the fat-loss group, **b** FVC in the fat-gain group, **c** FEV_1_ in the fat-loss group, **d** FEV_1_ in the fat-gain group, **e** FEV_1_/FVC in the fat-loss group, and **f** FEV_1_/FVC in the fat-gain group. Individual changes in FMI during follow-up were calculated with linear regression analysis. Participants with a slope of FMI change < 0 were classified under the fat-loss group, and those with a slope > 0 were classified under the fat-gain group. No participant had a zero-degree slope of FMI change throughout the study period. Individual changes in WHR during follow-up were calculated with linear regression analysis. Participants with a lower 30% of WHR change were designated to WHR-decreased group, and those with an upper 30% of WHR change were to WHR-increased group. WHR-stable group comprised the median 40% of participants, which included a zero-degree slope. Analysis was conducted in non-smoking women; age, height, residential area, follow-up duration, initial lung function, initial FMI, and initial WHR were adjusted. The gray shadow and numbers in parentheses represent 95% confidence intervals. *P-value between WHR-decreased and -increased group. *FEV*_*1*_ forced expiratory volume in 1 s, *FMI* fat mass index, *FVC* forced vital capacity, *WHR* waist-to-hip ratio

Compared with that in the WHR-decreased group, the lung function in the WHR-increased group prominently declined in both men and non-smoking women (Figs. 4, 5). In men, an increase in WHR was associated with a decline (WHR-decreased vs. WHR-increased groups) in FVC (fat-loss: − 25.9 mL/yr vs. − 35.8 mL/yr; fat-gain: − 35.8 mL/yr vs. − 46.3 mL/yr; all *P* < 0.001) and FEV_1_ (fat-loss: − 36.6 mL/yr vs. − 44.5 mL/yr; fat-gain: − 48.0 mL/yr vs. − 56.1 mL/yr; all *P* < 0.001)(Fig. 4). In non-smoking women, an increase in the WHR was also associated with a decline in FVC (fat-loss: − 24.3 mL/yr vs. − 32.5 mL/yr; fat-gain: − 26.7 mL/yr vs. − 38.4 mL/yr; all *P* < 0.001) and FEV_1_ (fat-loss: − 30.0 mL/yr vs. − 36.9 mL/yr; fat-gain: − 32.1 mL/yr vs. − 41.4 mL/yr; all *P* < 0.001) (Fig. 5). In Figs. 4 and 5, the 95% confidence intervals in FVC and FEV_1_ may overlap in early years due to small amount of lung volume change, but statistically significant differences in lung function change are evident at the end of follow-up.

Underweight participants (BMI < 18.5 kg/m^2^, n = 51) might have had illness-induced weight loss, or participants with severe obesity (BMI ≥ 30 kg/m^2^, n = 220) might have had other metabolic diseases [24, 25]. Therefore, we performed sensitivity analyses in participants with BMI between 18.5 and 30.0 kg/m^2^ (n = 4740; 2175 men, 2565 women). Additional file 5: Fig. S1 and Additional file 6: Fig. S2 represent the results, which are similar to those of the entire population.

## Discussion

In this study, we sought to determine the association between adiposity and lung function in the Asian general population. The cross-sectional analyses showed that higher adiposity was associated with lower lung function. During follow-up, an increase in the FMI was associated with a decline in FVC and FEV_1_ in both sexes, and an increased WHR was associated with a decline in FVC and FEV_1_ in men. Notably, participants in the WHR-increased group had a steeper decline in FVC and FEV_1_ than those in the WHR-decreased group, in both the fat-gain and fat-loss groups. Our findings suggest that changes in adiposity, especially central adiposity, strongly affect lung function in the middle-aged Asian general population.

Lung function impairment is associated with an increase in the incidence of chronic obstructive pulmonary disease [26, 27], cerebrovascular disease [28, 29], insulin resistance, diabetes [30], and all-cause mortality [31]. Obesity and being overweight are not only huge health burdens, but also affect lung function. Numerous cross-sectional studies [10, 11, 32] and a few longitudinal studies [12–14] have investigated the association between adiposity and lung function using BIA or dual-energy X-ray absorptiometry. However, previous studies have had some limitations, such as a small study population (47 women and 30 men) [12], narrow age range (32–38 years) [14], being limited to Western countries [12–14], and most importantly, having only two measurements, at the beginning and end of the study period [12–14]. In contrast to these studies, we grounded our analyses on a large-scale community-based cohort. More than 70% of the study population was followed up for 8 years, with ≥ 4 spirometry and anthropometric analyses. We used linear regression analysis to calculate the individual slopes of FMI and WHR changes. Through these comprehensive data analyses and linear mixed regression analyses, we demonstrated that increased adiposity was associated with decline in lung function. Moreover, this study excluded those with chronic lung diseases, such as chronic obstructive pulmonary disease or asthma. Therefore, our findings provide evident insights into the impact of central adiposity on lung function.

Central obesity is characterized by fat accumulation in the thorax, abdomen, and visceral organs. Fat deposition in the mediastinum and abdominal cavity reduces compliance of the respiratory system and changes the breathing pattern, resulting in a reduction in lung volumes, which is proportional to the severity of obesity [3]. In fact, expiratory flow velocity is determined by the degree of previous lung inflation through the elasticity of the lungs [33]. Therefore, decrease in lung volumes subsequently result in decrease in expiratory flow, which leads to a reduction in FEV_1_. Fat deposition also causes narrowing, closure, and hyperresponsiveness of the airway, thereby leading to gas trapping and ventilation inhomogeneity [3]. Accordingly, FMI and WHR were negatively associated with FVC and FEV_1_ in men in this study. Lung function decline was more prominent in the WHR-increased group, especially in the fat-gain group. Although the mechanism underlying the relationship between obesity and airway hyperresponsiveness remains to be established, the adipose tissue in individuals with obesity is infiltrated with activated macrophages interacting with adipocytes to induce systemic inflammation. Changes in adipose-derived inflammatory cytokines such as tumor necrosis factor-α, leptin, and adiponectin have the capacity to promote airway hyperresponsiveness [34].

In a study by Sutherland et al*.*, no longitudinal association was observed between body fat distribution and lung function in a 6-year follow-up in non-smoking, non-asthmatic young adults aged 32 and 38 years in New Zealand [14]. However, in our study, participants in the WHR-increased group tended to have more severe lung function impairment than those in the WHR-decreased group, in both the fat-loss and fat-gain groups. The older age of the participants in our study (40–69 years) might contribute to the difference. Age-related decline in FEV_1_ is estimated to be 25–30 mL/yr beginning at the age of 35–40 years, which increases to 60 mL/yr after the age of 70 years [35]. The age-adjusted decrease in lung function was greater in the WHR-increased group than in the WHR-decreased group, in both the fat-gain and fat-loss groups, suggesting that central obesity might have a more significant effect on lung function in the middle-aged population.

Additionally, although previous studies have reported improvements in lung function after weight loss in patients with obesity [36, 37], the fat-loss group with an increased WHR showed a more rapid lung function decline than those with a decreased WHR in this study. Therefore, our study clearly indicates that central obesity, not merely total adiposity, is the main driver for lung function impairment. Furthermore, in the South Korean general population, healthy never-smokers showed an FEV_1_ decline of 31.8 mL/yr and 27.0 mL/yr in men and women, respectively [38]. In comparison, the fat-gain group with an increased WHR showed the highest decline in FEV_1_ in both men (56.1 mL/yr) and women (41.4 mL/yr) in this study.

We noted sex-associated differences in this study as well; the annual rate of lung function decline was more prominent in men than in women. Consistent with our findings, Fenger et al. [13] and Sutherland et al. [14] also observed that the rate of lung function decline was more pronounced in men. A greater decline in lung function in men may suggest that lung function decline is directly proportional to lung size, due to the differences in airway caliber between males and females [13, 38].

According to our multiple linear mixed regression analysis (Table 2), FMI and WHR were both associated with lung function change in men, although FMI alone was associated with lung function change in women. Previous studies also have reported the different effects of fat and abdominal obesity on lung function decline between both sexes. The increase in waist circumference was related more prominently to FEV_1_ decline in men than in women [39]. In men, loss of fat mass over time was more closely associated with attenuated FEV_1_ reduction than the change in muscle mass [40]. Two possible mechanisms need to be considered. First, the mechanical effect of abdominal obesity affects differently to lung between the sexes. Abdominal and thoracic fat mass reduce the room for lung expansion, reducing vital capacity and limiting expiratory flow. As men have more abdominal fat than women when they have the same degree of adiposity, the mechanical aspect of central obesity in the respiratory system might be responsible for the sex-associated difference [13, 41]. Second, gain of adipose tissue may accentuate inflammatory processes, which can damage the alveolus and airway.

Additionally, the heavy smoking history in men compared with that in women might have been another reason. Different thresholds for detrimental effects of pulmonary irritants are expected between sexes [13]. Moreover, hormonal differences, mainly affected by menopause or hormonal replacement therapy, might have also contributed to the sex-associated difference [42]. Further comprehensive studies are needed to elucidate the sex-associated difference in lung function decline.

A few limitations of this study should be considered. First, BIA might not be as accurate as dual-energy X-ray absorptiometry since the former is influenced by hydration status and body morphology [43]. However, multiple studies have substantiated that the former is still a useful approach for assessing body composition with minimal errors in large epidemiological studies [11, 14, 44]. Second, pre-bronchodilator spirometry measurements alone were performed. However, these spirometry data were obtained under strict quality control. Third, we did not consider physical activities, aerobic fitness, dietary patterns, or chronic medical conditions such as diabetes, hypothyroidism, sleep apnea, neurological diseases, and long-term corticosteroid use, which could be possible confounders. Fourth, we divided participants into only two (fat loss and fat gain) or three (WHR-decreased, -stable, and -increased) groups, which could oversimplify the intensity of adiposity change. Finally, the cross-sectional analyses between adiposity and lung function at baseline showed a small correlation coefficient (Figs. 2 and 3). However, these are unadjusted results, and the longitudinal analyses after adjustment supported the importance of central adiposity change.

In conclusion, our study demonstrated that increased adiposity, especially central obesity, was associated with long-term impairment of lung function. An increase in FMI was associated with a significant decline in FVC and FEV_1_ in both sexes, whereas it was positively related to FEV_1_/FVC in women. However, an increase in the WHR was inversely associated with a decrease in FVC and FEV_1_ in men only. Moreover, the WHR-increased group showed a faster decline in FVC and FEV_1_ in both the fat-loss and fat-gain groups, suggesting that central obesity markedly reduces respiratory function in the middle-aged Asian general population.

## Supplementary Information

**Additional file 1: Table S1.** Number of participants in the Korean genome and epidemiology study.

**Additional file 2: Table S2.** Full model of multiple linear mixed regression analysis for long-term associations between adiposity indices and lung function in men. Full models of multiple linear mixed regression analysis in men.

**Additional file 3: Table S3.** Baseline characteristics according to adiposity changes in men. Subgroup analysis.

**Additional file 4: Table S4.** Baseline characteristics according to adiposity changes in women*. Subgroup analysis.

**Additional file 5: Figure S1.** Sensitivity analysis for lung function decline according to adiposity changes in Men with BMI between 18.5 and 30.0 kg/m^2^. Sensitivity analysis.

**Additional file 6: Figure S2.** Sensitivity analysis for lung function decline according to adiposity changes in non-smoking women with BMI between 18.5 and 30.0 kg/m^2^. Sensitivity analysis.

## Data Availability

The cohort data used in this study are publicly available.

## Abbreviations

BIA: Bio-electrical impedance analysis
BMI: Body mass index
FEV_1_: Forced expiratory volumes in 1 s
FMI: Fat mass index
FVC: Forced vital capacity
WHR: Waist-to-hip ratio
