# Supplementary material for: Longitudinal association between adiposity changes and lung function deterioration
Source: Respir Res. 2023 Feb 7;24:44. doi: 10.1186/s12931-023-02322-8 (PMC9903501; doi:10.1186/s12931-023-02322-8)
Supplement: Supplementary file 1 — Additional file 1: Table S1. Number of participants in the Korean genome and epidemiology study. [file 12931_2023_2322_MOESM1_ESM.doc]

**Table S1.** Number of Participants in the Korean Genome and Epidemiology Study

|  | Year | From Ansan (urban) | From Ansung (rural) | Total participants | Participants  in this study |
| --- | --- | --- | --- | --- | --- |
| Baseline | 2001-2002 | 5012 | 5018 | 10030 |  |
| 1^st^ follow-up | 2003-2004 | 4023 | 3540 | 7563 |  |
| 2^nd^ follow-up | 2005-2006 | 3540 | 3975 | 7515 | 5011 |
| 3^rd^ follow-up | 2007-2008 | 3255 | 3433 | 6688 | 4628 |
| 4^th^ follow-up | 2009-2010 | 3262 | 3403 | 6665 | 4232 |
| 5^th^ follow-up | 2011-2012 | 3052 | 3186 | 6238 | 4044 |
| 6^th^ follow-up | 2013-2014 | 3000 | 2906 | 5906 | 3847 |
