# Supplementary material for: Longitudinal association between adiposity changes and lung function deterioration
Source: Respir Res. 2023 Feb 7;24:44. doi: 10.1186/s12931-023-02322-8 (PMC9903501; doi:10.1186/s12931-023-02322-8)
Supplement: Supplementary file 2 — Additional file 2: Table S2. Full model of multiple linear mixed regression analysis for long-term associations between adiposity indices and lung function in men. Full models of multiple linear mixed regression analysis in men. [file 12931_2023_2322_MOESM2_ESM.doc]

**Table S2.** Full Model of Multiple Linear Mixed Regression Analysis for Long-term Associations Between Adiposity Indices and Lung Function in Men

|  | FVC, mL | | |  | FEV_1_, mL | | |  | FEV_1_/FVC, % | | |
| --- | --- | --- | --- | --- | --- | --- | --- | --- | --- | --- | --- |
|  | Estimated | SE | P-value |  | Estimated | SE | P-value |  | Estimated | SE | P-value |
| **Model 1** |  |  |  | **Model 2** |  |  |  | **Model 3** |  |  |  |
| Age, yr | -4.2 | 1.1 | <0.001 | Age, yr | -5.3 | 1.0 | <0.001 | Age, yr | -0.03 | 0.02 | 0.125 |
| Height, cm | 2.7 | 0.6 | <0.001 | Height, cm | 1.6 | 0.5 | <0.001 | Height, cm | -0.00 | 0.01 | 0.672 |
| Time, yr | -36.1 | 0.6 | <0.001 | Time, yr | -47.1 | 0.5 | <0.001 | Time, yr | -0.46 | 0.01 | <0.001 |
| Pack-yr | -0.1 | 0.2 | 0.687 | Pack-yr | -0.3 | 0.1 | 0.033 | Pack-yr | -0.01 | 0.00 | 0.023 |
| Area - Urban | -73.2 | 6.2 | <.001 | Area - Urban | -22.9 | 5.2 | <0.001 | Area - Urban | 0.88 | 0.09 | <0.001 |
| Initial FVC, L | 910.1 | 6.0 | <0.001 | Initial FEV_1_, L | 907.8 | 6.2 | <0.001 | Initial FEV_1_/FVC | 0.93 | 0.01 | <0.001 |
| FMI, kg/m^2^ | -31.8 | 10.5 | 0.002 | FMI, kg/m^2^ | -38.2 | 8.8 | <0.001 | FMI, kg/m^2^ | -0.04 | 0.15 | 0.775 |
| FMI * Age | 0.2 | 0.2 | 0.414 | FMI * Age | 0.4 | 0.2 | 0.013 | FMI * Age | 0.00 | 0.00 | 0.685 |
| **Model 4** |  |  |  | **Model 5** |  |  |  | **Model 6** |  |  |  |
| Age, yr | -18.4 | 5.0 | <0.001 | Age, yr | -12.6 | 4.1 | 0.002 | Age, yr | 0.13 | 0.07 | 0.082 |
| Height, cm | 2.9 | 0.6 | <0.001 | Height, cm | 1.9 | 0.5 | <0.001 | Height, cm | -0.00 | 0.01 | 0.597 |
| Time, yr | -36.8 | 0.6 | <0.001 | Time, yr | -47.4 | 0.5 | <0.001 | Time, yr | -0.46 | 0.01 | <0.001 |
| Pack-yr | 0.0 | 0.2 | 0.858 | Pack-yr | -0.2 | 0.1 | 0.061 | Pack-yr | -0.01 | 0.00 | 0.027 |
| Area - Urban | -101.9 | 6.4 | <.001 | Area - Urban | -45.2 | 5.4 | <0.001 | Area - Urban | 0.88 | 0.10 | <0.001 |
| Initial FVC, L | 920.4 | 5.9 | <0.001 | Initial FEV_1_, L | 913.2 | 6.1 | <0.001 | Initial FEV_1_/FVC | 0.93 | 0.01 | <0.001 |
| WHR | -1242.2 | 290.7 | <.001 | WHR | -849.8 | 241.8 | <0.001 | WHR | 8.30 | 4.31 | 0.054 |
| WHR * Age | 16.7 | 5.3 | 0.002 | WHR * Age | 10.5 | 0.004 | 0.018 | WHR * Age | -0.16 | 0.08 | 0.044 |

Note: Models were adjusted for age, height, residential area, follow-up duration, smoking exposure in pack-years, initial adiposity indices, interaction between age and adiposity indices, and initial lung function.

Abbreviations: FEV_1_, forced expiratory volume in 1 s; FMI, fat mass index; FVC, forced vital capacity; SE, standard error; WHR, waist-to-hip ratio
