# Supplementary material for: Longitudinal association between adiposity changes and lung function deterioration
Source: Respir Res. 2023 Feb 7;24:44. doi: 10.1186/s12931-023-02322-8 (PMC9903501; doi:10.1186/s12931-023-02322-8)
Supplement: Supplementary file 3 — Additional file 3: Table S3. Baseline characteristics according to adiposity changes in men. Subgroup analysis. [file 12931_2023_2322_MOESM3_ESM.doc]

**Table S3.** Baseline Characteristics According to Adiposity Changes in Men^*^

|  |  | Fat loss^†^ (n = 764^‡^) | | | |  | Fat gain^†^ (n = 1490) | | | |
| --- | --- | --- | --- | --- | --- | --- | --- | --- | --- | --- |
|  | WHR-  decreased^§^  (n = 360) | | WHR-  stable^§^  (n = 278) | WHR-  increased^§^ (n = 126) | *P-*value | WHR-  decreased^§^ (n = 316) | | WHR-  stable^§^  (n = 624) | WHR -increased^§^ (n = 550) | *P-*value |
| Age, years | 54.0 ± 7.5 | | 52.3 ± 6.7 | 52.3 ± 7.1 | .006 | 54.9 ± 8.1 | | 52.9 ± 7.4 | 52.4 ± 7.2 | <.001 |
| Height, cm | 167.2 ± 5.4 | | 168.1 ± 5.7 | 166.8 ± 5.5 | .042 | 166.8 ± 6.0 | | 167.4 ± 5.8 | 167.5 ± 5.6 | .210 |
| BMI, kg/m^2^ | 24.8 ± 2.8 | | 24.9 ± 2.6 | 24.8 ± 2.7 | .663 | 24.4 ± 2.8 | | 24.5 ± 2.7 | 24.3 ± 2.6 | .615 |
| Ever smoker | 269 (74.7) | | 204 (73.4) | 87 (69.0) | .464 | 225 (71.2) | | 461 (73.9) | 412 (74.9) | .486 |
| Smoking exposure, pack-years | 18.6 ± 20.0 | | 18.0 ± 18.3 | 17.9 ± 21.3 | .909 | 20.1 ± 20.3 | | 18.4 ± 17.3 | 19.1 ± 17.6 | .386 |
| Residential area – rural | 152 (42.2) | | 55 (19.8) | 25 (19.8) | <.001 | 205 (64.9) | | 274 (43.9) | 126 (22.9) | <.001 |
| Residential area – urban | 208 (57.8) | | 223 (80.2) | 101 (80.2) |  | 111 (35.1) | | 350 (56.1) | 424 (77.1) |  |
| Adiposity index |  | |  |  |  |  | |  |  |  |
| FMI, kg/m^2^ | 5.6 ± 1.7 | | 5.7 ± 1.6 | 5.6 ± 1.7 | .687 | 5.1 ± 1.7 | | 5.1 ± 1.6 | 5.1 ± 1.6 | .903 |
| WHR | 0.93 ± 0.06 | | 0.91 ± 0.05 | 0.90 ± 0.05 | <.001 | 0.95 ± 0.06 | | 0.92 ± 0.05 | 0.90 ± 0.05 | <.001 |
| Abdominal obesity^ll^ | 255 (70.8) | | 168 (60.4) | 64 (50.8) | <.001 | 242 (76.8) | | 416 (66.8) | 254 (46.4) | <.001 |
| Respiratory function |  | |  |  |  |  | |  |  |  |
| FVC, L | 4.20 ± 0.63 | | 4.26 ± 0.61 | 4.24 ± 0.61 | .470 | 4.17 ± 0.67 | | 4.29 ± 0.62 | 4.31 ± 0.63 | .006 |
| FVC, % predicted | 101.2 ± 12.0 | | 100.4 ± 11.8 | 101.6 ± 11.3 | .576 | 101.7 ± 12.4 | | 102.4 ± 11.5 | 102.5 ± 11.5 | .606 |
| FEV_1_, L | 3.31 ± 0.53 | | 3.38 ± 0.50 | 3.35 ± 0.49 | .209 | 3.30 ± 0.56 | | 3.37 ± 0.51 | 3.41 ± 0.52 | .012 |
| FEV_1_, % predicted | 107.9 ± 13.6 | | 107.3 ± 13.2 | 108.0 ± 12.5 | .807 | 109.1 ± 14.2 | | 108.7 ± 12.9 | 108.9 ± 12.7 | .924 |
| FEV_1_/FVC | 78.7 ± 4.9 | | 79.3 ± 4.5 | 79.1 ± 4.9 | .250 | 78.9 ± 5.0 | | 78.8 ± 4.8 | 79.0 ± 4.8 | .704 |

^*^Data are presented as numbers (%) or means ± standard deviations.

^†^Individual changes in FMI during follow-up were calculated with linear regression analysis. Participants with a slope of FMI change < 0 were classified under the fat-loss group, and those with a slope > 0 were classified under the fat-gain group. No participant had a zero-degree slope of FMI change throughout the study period.

^‡^One individual was omitted due to missing data on baseline WHR.

^§^Individual changes in WHR during follow-up were calculated with linear regression analysis. We divided study participants with a slope of WHR change. Participants with a lower 30% of WHR change were designated to WHR-decreased group, and those with an upper 30% of WHR change were to WHR-increased group. WHR-stable group comprised the median 40% of participants, which included a zero-degree slope.

^ll^Abdominal obesity in men was defined by a WHR ≥ 0.90.

Abbreviations: BMI, body mass index; FEV_1_, forced expiratory volume in 1 s; FMI, fat mass index; FVC, forced vital capacity; WHR, waist-to-hip ratio
