# Supplementary material for: Longitudinal association between adiposity changes and lung function deterioration
Source: Respir Res. 2023 Feb 7;24:44. doi: 10.1186/s12931-023-02322-8 (PMC9903501; doi:10.1186/s12931-023-02322-8)
Supplement: Supplementary file 4 — Additional file 4: Table S4. Baseline characteristics according to adiposity changes in women*. Subgroup analysis. [file 12931_2023_2322_MOESM4_ESM.doc]

**Table S4.** Baseline Characteristics According to Adiposity Changes in Women^*^

|  |  | Fat loss^†^ (n = 969) | | | |  | Fat gain^†^ (n = 1787) | | |
| --- | --- | --- | --- | --- | --- | --- | --- | --- | --- |
|  | WHR-  decreased^‡^ (n = 400) | | WHR-  stable^‡^ (n = 378) | WHR-increased^‡^ (n = 191) | *P-*value | WHR-decreased^‡^ (n = 427) | WHR-  stable^‡^ (n = 724) | WHR-  increased^‡^ (n = 636) | *P-*value |
| Age, years | 57.9 ± 8.7 | | 54.5 ± 8.0 | 54.3 ± 8.0 | <.001 | 57.1 ± 8.4 | 53.6 ± 7.7 | 52.9 ± 7.8 | <.001 |
| Height, cm | 153.4 ± 5.5 | | 154.5 ± 5.2 | 154.8 ± 5.7 | .004 | 153.4 ± 5.8 | 154.6 ± 5.3 | 154.9 ± 5.4 | <.001 |
| BMI, kg/m^2^ | 25.4 ± 3.1 | | 25.1 ± 3.0 | 24.9 ± 3.0 | .253 | 24.8 ± 3.2 | 24.7 ± 3.1 | 24.2 ± 3.1 | .003 |
| Ever smoker | 7 (1.8) | | 9 (2.4) | 4 (2.1) | .825 | 11 (2.6) | 16 (2.2) | 21 (3.3) | .456 |
| Smoking exposure, pack-years | 0.3 ± 2.8 | | 0.1 ± 1.2 | 0.2 ± 2.1 | .408 | 0.4 ± 3.4 | 0.1 ± 1.3 | 0.5 ± 3.7 | .089 |
| Residential area – rural | 233 (58.2) | | 132 (34.9) | 34 (17.8) | <.001 | 330 (77.3) | 393 (54.3) | 176 (27.7) | <.001 |
| Residential area – urban | 167 (41.8) | | 246 (65.1) | 157 (82.2) |  | 97 (22.7) | 331 (45.7) | 460 (72.3) |  |
| Adiposity index |  | |  |  |  |  |  |  |  |
| FMI, kg/m^2^ | 8.3 ± 2.1 | | 8.2 ± 2.1 | 8.1 ± 2.0 | .666 | 7.7 ± 2.3 | 7.6 ± 2.2 | 7.4 ± 2.1 | .055 |
| WHR | 0.94 ± 0.08 | | 0.89 ± 0.08 | 0.86 ± 0.06 | <.001 | 0.96 ± 0.08 | 0.90 ± 0.08 | 0.85 ± 0.07 | <.001 |
| Abdominal obesity^§^ | 389 (97.2) | | 333 (88.3) | 158 (83.2) | <.001 | 417 (97.7) | 648 (89.8) | 489 (77.0) | <.001 |
| Respiratory function |  | |  |  |  |  |  |  |  |
| FVC, L | 2.88 ± 0.51 | | 3.04 ± 0.49 | 3.04 ± 0.50 | <.001 | 2.88 ± 0.49 | 3.03 ± 0.49 | 3.10 ± 0.50 | <.001 |
| FVC, % predicted | 106.5 ± 13.0 | | 107.1 ± 12.0 | 106.7 ± 13.0 | .785 | 106.2 ± 12.9 | 105.9 ± 12.6 | 107.1 ± 12.9 | .240 |
| FEV_1_, L | 2.33 ± 0.43 | | 2.48 ± 0.41 | 2.48 ± 0.41 | <.001 | 2.34 ± 0.42 | 2.47 ± 0.41 | 2.53 ± 0.42 | <.001 |
| FEV_1_, % predicted | 117.5 ± 15.4 | | 117.5 ± 14.9 | 116.6 ± 15.4 | .756 | 117.0 ± 15.7 | 115.6 ± 14.7 | 116.4 ± 14.8 | .264 |
| FEV_1_/FVC | 81.1 ± 4.6 | | 81.8 ± 4.5 | 81.6 ± 4.5 | .096 | 81.2 ± 4.7 | 81.6 ±4.5 | 81.5 ± 4.4 | .306 |

^*^Data are presented as numbers (%) or means ± standard deviations.

^†^Individual changes in FMI during follow-up were calculated with linear regression analysis. Participants with a slope of FMI change < 0 were classified under the fat-loss group, and those with a slope > 0 were classified under the fat-gain group. No participant had a zero-degree slope of FMI change throughout the study period.

^‡^Individual changes in WHR during follow-up were calculated with linear regression analysis. We divided study participants with a slope of WHR change. Participants with a lower 30% of WHR change were designated to WHR-decreased group, and those with an upper 30% of WHR change were to WHR-increased group. WHR-stable group comprised the median 40% of participants, which included a zero-degree slope.

^§^Abdominal obesity in women was defined by WHR ≥ 0.80.

Abbreviations: BMI, body mass index; FEV_1_, forced expiratory volume in 1 s; FMI, fat mass index; FVC, forced vital capacity; WHR, waist-to-hip ratio
