# Supplementary material for: Longitudinal association between adiposity changes and lung function deterioration
Source: Respir Res. 2023 Feb 7;24:44. doi: 10.1186/s12931-023-02322-8 (PMC9903501; doi:10.1186/s12931-023-02322-8)

**Figure S1.** Sensitivity analysis for lung function decline according to adiposity changes in men with BMI between 18.5 kg/m^2^ and 30.0 kg/m^2^

Lung function declines were compared between the WHR-decreased, WHR-stable, and WHR-increased groups in fat-loss and fat-gain group, respectively. Decline of (**a**) FVC in the fat-loss group, (**b**) FVC in the fat-gain group, (**c**) FEV_1_ in the fat-loss group, (**d**) FEV_1_ in the fat-gain group, (**e**) FEV_1_/FVC in the fat-loss group, and (**f**) FEV_1_/FVC in the fat-gain group.

Individual changes in FMI during follow-up were calculated with linear regression analysis. Participants with a slope of FMI change < 0 were classified under the fat-loss group, and those with a slope > 0 were classified under the fat-gain group. No participant had a zero-degree slope of FMI change throughout the study period.

Individual changes in WHR during follow-up were calculated with linear regression analysis. Participants with a lower 30% of WHR change were designated to WHR-decreased group, and those with an upper 30% of WHR change were to WHR-increased group. WHR-stable group comprised the median 40% of participants, which included a zero-degree slope.

Age, height, residential area, follow-up duration, smoking exposure (pack-years), initial lung function, initial FMI, and initial WHR were adjusted. The grey shadow and numbers in parentheses represent 95% confidence intervals.

*P-value between WHR-decreased and -increased group

Abbreviations: BMI, body mass index; FEV_1_, forced expiratory volume in 1 s; FMI, fat mass index; FVC, forced vital capacity; WHR, waist-to-hip ratio


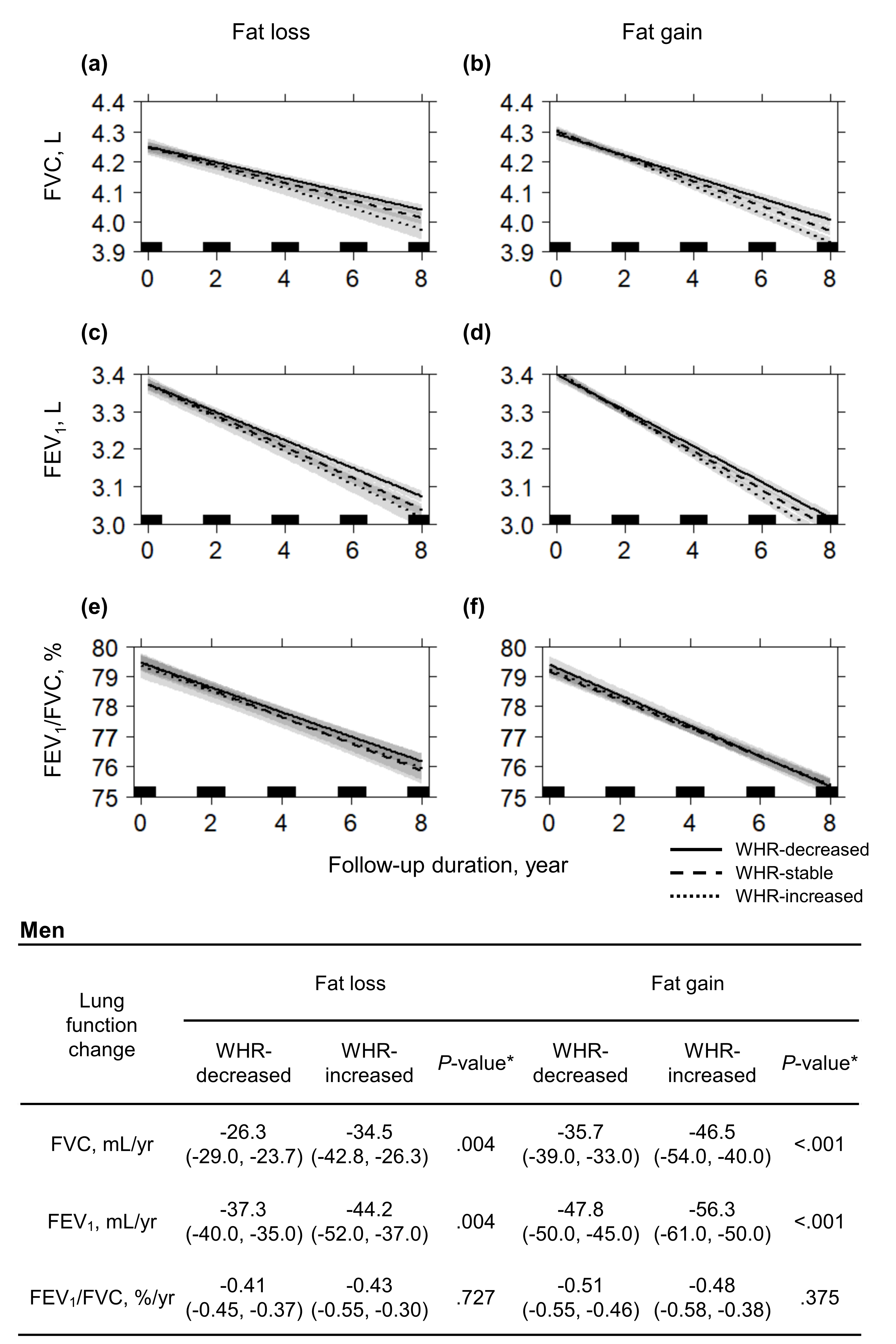

Supplement: Supplementary file 5 — Additional file 5: Figure S1. Sensitivity analysis for lung function decline according to adiposity changes in Men with BMI between 18.5 and 30.0 kg/m2. Sensitivity analysis. [file 12931_2023_2322_MOESM5_ESM.doc]
